# Incidence of post myocardial infarction left ventricular thrombus formation in the era of primary percutaneous intervention and glycoprotein IIb/IIIa inhibitors. A prospective observational study

**DOI:** 10.1186/1476-7120-4-20

**Published:** 2006-04-06

**Authors:** Arshad Rehan, Manpreet Kanwar, Howard Rosman, Sujood Ahmed, Arshad Ali, Julius Gardin, Gerald Cohen

**Affiliations:** 1 Department of Cardiology, St John Hospital and Medical Centre, Wayne State University, 22101 Moross Road, Detroit, Michigan 48230, USA; 2 Department of Cardiology, Guthrie Clinic Sayre, Guthrie Square, Sayer, Pennsylvania 18840, USA

## Abstract

**Background:**

Before the widespread use of primary percutaneous coronary intervention (PCI) and glycoprotein IIb/IIIa inhibitors (GP IIb/IIIa) left ventricular (LV) thrombus formation had been reported to complicate up to 20% of acute myocardial infarctions (AMI). The incidence of LV thrombus formation with these treatment modalities is not well known.

**Methods:**

92 consecutive patients with ST-elevation AMI treated with PCI and GP IIb/IIIa inhibitors underwent 2-D echocardiograms, with and without echo contrast agent, within 24–72 hours.

**Results:**

Only 4/92 (4.3%) had an LV thrombus, representing a significantly lower incidence than that reported in the pre-PCI era. Use of contrast agents did not improve detection of LV thrombi in our study.

**Conclusion:**

The incidence of LV thrombus formation after acute MI, in the current era of rapid reperfusion, is lower than what has been historically reported.

## Background

A well-recognized complication of acute myocardial infarction (AMI) is the development of a left ventricular (LV) thrombus. Causes of LV thrombus include segmental dysfunction of the infarcted myocardium causing stasis, endocardial tissue inflammation providing a thrombogenic surface, and a hypercoagulable state [1-6]. There is evidence that LV thrombi usually develop within a few days after AMI [2,7-9].

Historically, the incidence of LV thrombi complicating AMI had been reported to be 20–40%, and may reach 60% among patients with large anterior wall AMI [10]. Early thrombolytic therapy reduces this incidence [5,6,11]. However, there is little data on the incidence of LV thrombus formation after primary percutaneous coronary intervention (PCI), with concurrent use of IIb/IIIa inhibitors, for AMI. We hypothesized that with improved reperfusion using catheter-based techniques, together with the use of potent platelet glycoprotein IIb/IIIa inhibitor therapy [12], the incidence of post AMI LV thrombus formation would be lower than what had been reported in the pre-PCI era. To increase the sensitivity of standard two-dimensional echocardiography (2-D echo) for detection of an LV thrombus, we used a third-generation contrast agent to outline the LV cavity structures.

## Methods

Ninety-two consecutive patients presenting to our institution with ST elevation AMI and treated with PCI, rescue angioplasty after failed thrombolysis, or 'facilitated' PCI were enrolled in the study. Written informed consent was obtained from the patients prior to enrolment. Baseline demographic characteristics, pre- and post-intervention Thrombolysis in Myocardial Infarction flow grade, type of intervention, and other therapies instituted were recorded. Two-dimensional echocardiography was performed using a Vivid-7 ultrasound machine (GE Medical Systems) within three days of the PCI, by a registered sonographer, with and without an echo contrast agent (Perflutren Lipid Microspheres – Definity^®^, Bristol-Myers Squibb Inc), with digital storage for later off-line analysis. Second harmonic imaging was used to optimise endocardial visualization. Two level-3 echocardiographers blinded to the clinical details separately reviewed the echo images in each patient. The contrast images were stored and reviewed separately from the non-contrast images. LV thrombus was defined as an echodense mass with definite margins, contiguous but distinct from the endocardium, adjacent to an area of hypo- or akinetic myocardium [1]. In cases where there was a difference of interpretation between the two readers, both readers reviewed the images together and came to a consensus. A single reader, blinded to the clinical and 2-D echo details of the patients reviewed the angiographic data.

## Results

Fifty-seven men and 35 women were studied, with a mean age of 60 years (range: 30 – 87 years) (Table 1). Forty-one patients had an inferior MI involving the right coronary artery, 37 had an anterior MI with the left anterior descending artery as the culprit vessel, 5 patients had an infarct related to the left circumflex, 4 had an acute occlusion of an obtuse marginal branch, 3 had occluded vein grafts and one each had occlusion of the ramus intermedius branch and the major diagonal branch. All patients had ST elevation on their presenting electrocardiograms. Eight patients underwent PCI for failed thrombolysis and 10 had 'facilitated' PCI after administration of half-dose thrombolytics. All but 5 patients received glycoprotein IIb/IIIa inhibitor therapy. Stents were deployed in 78 patients, whereas 14 patients had angioplasty alone. Only 1 patient failed to achieve TIMI III flow in the infarct related vessel.

**Table 1:** Baseline demographics

	**n(%) (Total n = 92)**
Mean Age in Years (Range)	59 (30–87)
Males	57 (62%)
History of coronary disease	17 (18%)
Hypertension	47 (51%)
Congestive Heart Failure	2 (2%)
Diabetes Mellitus	22 (24%)
Medications on admission	
- Beta-Blocker	19 (21%)
- ACEI	9 (10%)
- Aspirin	22 (24%)
- Clopidogrel	0
- Warfarin	1 (1%)
- Statins	14 (15%)

n: Number

% denotes percentage of each characteristic among total patients

ACEI: Angiotensin Converting Enzyme Inhibitor

**Figure 1:**
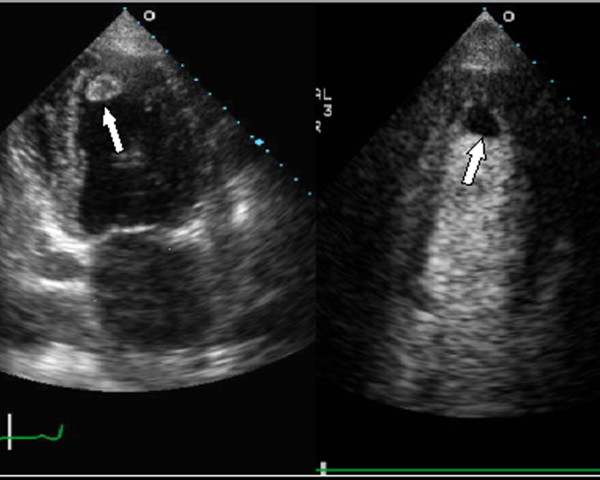
**Thrombus in left ventricular apex**. Thrombus noted in left ventricular apex (white area in non-contrast enhanced image on left, black lucent area in contrast enhanced image on right)

Four patients, all with an anterior MI (4.3% of the total, 10.8 % of the anterior MI group), had a definite LV thrombus, both on unenhanced and enhanced imaging with perflutren lipid microspheres (Table 2). The thrombus was located in the LV apex in all 4 cases (Figure 1). All 4 were men (Table 3), with a mean age of 71 years (range 52 – 81 years); all demonstrated an occlusion of the proximal or mid LAD, and achieved TIMI III flow after PCI. The mean LV ejection fraction was 31% (range 20–40%). None of the 4 had a prior history of coronary artery disease. Three of these patients had undergone primary PCI and received IIb/IIIa therapy, whereas one patient, who did not receive GP IIb/IIIa inhibitor therapy, underwent rescue PCI after failing thrombolytic therapy. Echo contrast agent did not reveal LV thrombus in any patient where one was not seen on routine, unenhanced 2-D echocardiogram.

**Table 2:** Relationship between infarct location and thrombus formation

**Infarct Location**	**Number of Patients**	**LV Thrombus n (%)**
Anterior	37	4 (10.8)
Inferior	42	0
Posterior-Inferior	12	0
Lateral	1	0
**Total**	**92**	**4 (4.3)**

n: Number

LV = Left ventricular

% represents percentage of a characteristic within each category

**Table 3:** Features of patients with left ventricular thrombi

	**Patient 1**	**Patient 2**	**Patient 3**	**Patient 4**
Age (years)	68	81	52	81
Sex	Male	Male	Male	Male
IRA	Prox LAD	Prox LAD	Mid LAD	Prox LAD
Lytics	No	No	No	Yes (failed)
PCI	Stent	Stent	Stent	Stent
IIb/IIIa	Yes	Yes	Yes	No
EF	23%	32%	35%	30%

IRA = Infarct related artery

LAD = Left Anterior Descending artery, Prox = Proximal

PCI = Percutaneous Intervention

EF = Ejection Fraction

## Discussion

Previous studies have shown that mural thrombi occur in approximately 20% of all patients who do not receive reperfusion therapy [10]. This incidence rises to 40% in case of anterior AMI and further to 60% in cases of large anterior AMI involving the LV apex10. Patients with LV thrombi have a worse overall prognosis [13], with about 10% thrombi resulting in systemic embolization [10]. Strategies to prevent this complication therefore represent an important therapeutic goal.

Although the introduction of thrombolytic therapy for treatment of AMI improved survival, its impact on the incidence of LV thrombus formation has varied. Bhatnagar, et-al showed a 3-fold reduction in the incidence of LV thrombus in patients receiving early intravenous recombinant tissue plasminogen activator [5]. A sub study of the Gruppo Italiano per lo Studio della Sopravvivenza nell'infarto miocardico (GISSI-2), however, failed to show a significant reduction in the incidence of LV thrombus formation in AMI patients with thrombolytic agents [14]. An analysis of the subsequent GISSI-3 database by Chiarella et al, however, showed a significant reduction in the incidence of LV thrombus in patients with AMI [15]. In a meta-analysis of 6 studies, Vaitkus and Barnathan found an association between thrombolytic therapy and reduced LV thrombus formation, although it did not achieve statistical significance [11].

Catheter-based reperfusion therapy is superior to thrombolytics in promoting early myocardial recovery, with improved clinical outcomes [12]. Primary PCI, with or without stenting, has thus become the treatment of choice for patients with AMI in institutions with facilities for emergency cardiac catheterization [16,17]. The introduction of potent glycoprotein IIb/IIIa (GP IIb/IIIa) inhibitor agents has further improved the procedural outcomes for PCI with stenting in AMI [12,18,19]. In one small study, the incidence of LV thrombus formation after AMI in patients undergoing primary PCI, was reported to be as low as to 4% [20]. Porter et al, in a retrospective study of AMI patients who received either thrombolytics or primary PCI, with or without GP IIb/IIIa inhibitors, reported a 23.5% incidence of LV thrombus after anterior AMI [21]. Our study is unique in that it prospectively evaluates LV thrombus formation in AMI patients receiving both primary PCI and GP IIb/IIIa inhibitors. It confirms a significant reduction in this complication that parallels improvement in other post-MI outcomes as a result of better contemporary therapy. We also used echo contrast agent in all patients and found no additive value in detection of post MI LV thrombi.

It is important to review the role of echocardiography in the diagnosis of LV thrombi. The sensitivity and specificity of 2-D echo in the diagnosis of LV thrombi has been established, even with first and second-generation echo equipment, to be in excess of 92% and 86–88%, respectiviely1. With modern, improved imaging equipment, the sensitivity is expected to be even higher. Nonetheless 2-D echo has certain limitations. First, a small thrombus (e.g., < 5–6 mm) may not be accurately detected [22]. Second, differences in acoustic impedance between endocardium and freshly formed thrombus may not be sufficient to allow clear definition of the thrombus. Third, various other anatomic structures, such as false tendons or trabeculae, may confound the diagnosis [1].

The use of contrast agents has been reported to add to the sensitivity of non-contrast 2-D echo [23] in the diagnosis of LV thrombi, although in our study, contrast agents did not detect any additional thrombi. The use of contrast adds US $110 to the cost of each study and involves approximately 5 minutes of additional imaging time. Perhaps it would be reasonable to reserve contrast agents for patients with sub-optimal 2D imaging which precludes adequate visualization of the endocardium. Given the small number of patients with thrombi in our study the additive value of contrast should be interpreted with caution.

## Conclusion

In conclusion, the incidence of early LV thrombus formation is lower (4% for all MI's, 11% for anterior MI's) in this era of primary PCI, coupled with the use of potent antithrombotic and anti-platelet agents, compared to historical data in the pre-PCI era (up to 20 % for all MI's, 40% for anterior MI's). Routine use of echo contrast agents did not improve detection of LV thrombi in our study.

## Competing interests

The author(s) declare that they have no competing interests.

## Authors' contributions

AR conceived the study and drafted the manuscript

MK recruited patients and assisted in data collection

HR analysed the echocardiographic images and made important revisions to the manuscript

SA participated in acquisition and analysis of the data.

AA participated in design and coordination of the study and interpreted the angiograms

JG made important intellectual contributions to the manuscript

GC worked on data analysis and interpretation of echocardiograms, and made important contributions to the manuscript
